# Optimization of meropenem dosing regimens in critically ill patients with augmented renal clearance

**DOI:** 10.3389/fmed.2025.1550053

**Published:** 2025-05-09

**Authors:** Jinfeng Luo, Jing Liu, Hongfu Lin, Yang Yang, Caihong Chen, Jianping Chen, Han Zhong, Shipao Zhang

**Affiliations:** ^1^Department of Cardiology, The Second Hospital of Sanming, Sanming, Fujian, China; ^2^Department of Critical Care Medicine, The Second Hospital of Sanming, Sanming, Fujian, China; ^3^Department of Nephrology, The Second Hospital of Sanming, Sanming, Fujian, China; ^4^Department of Pharmacy, Renji Hospital, School of Medicine, Shanghai Jiao Tong University, Shanghai, China

**Keywords:** meropenem, Monte Carlo simulation, augmented renal clearance, pharmacokinetics/pharmacodynamics, sepsis

## Abstract

The pharmacokinetics of meropenem are significantly altered in patients with augmented renal clearance (ARC), resulting in suboptimal plasma concentrations. The objective of this study is to investigate the efficacy of different meropenem regimens in critically ill patients with ARC. To this end, Monte Carlo simulations were conducted. The probability of target attainment (PTA) and the cumulative fraction of response (CFR) were evaluated with consideration of the minimal inhibitory concentration (MIC) breakpoint according to the Clinical and Laboratory Standards Institute (CLSI). The findings of this study demonstrate that meropenem administered at a dosage of 2 *g* every 8 h (q8 h) 2/3 h to critically ill patients with ARC [creatinine clearance (CrCL) of 140–200 mL/min] results in ≥ 90% PTA (100% *f*T > MIC) for lower MICs (≤ 2 mg/L). However, for higher MICs (4–8 mg/L), the administration of intensified regimens (2 g q8 h 4/6 h or continuous infusion) was necessary. The CFR analysis confirmed ≥ 90% target attainment for *Klebsiella pneumoniae* with regimens meropenem 2 g q8 h 2–6 h or continuous infusion, but not for *Acinetobacter baumannii* or *Pseudomonas aeruginosa*, regardless of regimen. For resistant *Klebsiella pneumoniae* (4 < MIC ≤ 8), prolonged (4–6 h) or continuous infusions are recommended. For *Acinetobacter baumannii* and *Pseudomonas aeruginosa*, alternative or combination therapies are advised due to insufficient PK/PD target attainment with meropenem monotherapy. The findings emphasize the importance of individualized dosing strategies in ARC patients, considering meropenem’s distinctive PK/PD characteristics, the pathogen’s MIC, and renal function, in order to effectively manage resistant Gram-negative infections while optimizing clinical outcomes.

## Introduction

Severe infection stands as a leading cause of intensive care unit (ICU) admission (1), the mortality of patients with such infection remains substantial (2). According to WHO data, infectious disease represents one of the top 10 causes of death worldwide (3). In American, more than 350,000 patients die from serious infections in a year (4). The significance of promptly initiating tailored treatments in critically ill patients with severe infections, particularly sepsis, is a matter of great concern (5). However, the pharmacokinetics (PK) of drugs can be altered by supportive technology and pathological processes in critically ill patients (6, 7). The routine dosage of anti-infective regimens may be insufficient to attain target plasma concentrations (8). Consequently, antimicrobial drug monitoring and dosage optimization are essential to achieve aggressive pharmacodynamics (PD) targets (9).

Gram-negative bacteria (GNB) are the primary pathogens identified in severe infections (10). Meropenem, a broad-spectrum carbapenem antibiotic that prevents the synthesis of essential components of the bacterial cell wall, resulting in the death of the microorganism, is an important treatment for severe GNB infections (11). For meropenem, a β-lactam antibiotic, the duration of time (T) that the unbounded drug concentration above the minimal inhibitory concentration (MIC) is the most important indicator, which is defined as *f*T > MIC (12). In the context of critically ill patients, the PK/PD target for β-lactam antibiotics is delineated as 100% *f*T > MIC (or more ambitiously, 100% *f*T > 4 × MIC), with the objective of enhancing survival rates and mitigating resistance (13, 14). Patients with 100% *f*T > MIC exhibited significantly higher rates of clinical cure (82%) and bacteriological eradication (97%) in comparison to patients with % *f*T > MIC less than 100% (15). Consequently, in the present study, 100% *f*T > MIC were utilized as PK/PD targets for Monte Carlo simulation.

Augmented renal clearance (ARC) is a pathophysiological phenomenon that often occurs in critically ill patients, resulting in enhanced renal function defined as a urinary creatinine clearance of at least 130 mL/min/1.73 m^2^ (16). The incidence of ARC in critically ill patients ranges from 30% to 65%, and can be as high as 50%–85% in those with sepsis, trauma, and other factors (17, 18). The mechanism of ARC is that the hyperdynamic and hypermetabolic state of critically ill patients increases cardiac output and renal blood flow, leading to increased drug clearance through the kidney (19). It has been reported that patients with ARC are less likely to achieve % *f*T > MIC with beta-lactam antibiotics (20). Udy et al. (21) also reported that only one third of critically ill patients with sepsis achieved 100% *f*T > MIC when using piperacillin-tazobactam, owing to elevated drug clearance. These results suggest that ARC promotes drug excretion and leads to inadequate drug exposure, which may compromise clinical efficacy.

In the present study, we aim to explore the alternative dosage regimens of meropenem in critically ill patients with ARC using Monte Carlo simulations. This will provide a potential recommendation for the development of antimicrobial outcomes for such patients.

## Materials and methods

### Monte Carlo simulations

Monte Carlo simulation was performed using Oracle Crystal Ball 11.1.2.4.850 software embedded in Office Excel 2019. Pharmacokinetic parameters including renal clearance (CL) and volume of distribution (Vd) were assumed to follow a normal distribution, while MIC followed a discrete uniform distribution and free drug fraction (*f*) followed a uniform distribution. The MIC value was set to a range of 0.125–8 μg/mL. A target value of 100% *f*T > MIC was set and different creatinine clearance (CrCL) values (140, 160, 180, and 200 mL/min) were tested. The probability of target attainment (PTA) value was then calculated using Monte Carlo simulations run for 10,000 cases for different meropenem dosing regimens as follows:

a: 1 g infused over 0.5 h every 8 h, 1 g q8 h 0.5 h;
b: 2 g infused over 2 h every 8 h, 2 g q8 h 2 h;
c: 2 g over 3 h every 8 h, 2 g q8 h 3 h;
d: 2 g over 4 h every 8 h, 2 g q8 h 4 h;
e: 2 g over 6 h every 8 h, 2 g q8 h 6 h;
f: 2 g over 8 h every 8 h, continuous infusion.

The results were plotted as PTA-MIC curves.

Equation 1 (22) was used to calculate the values of % *f*T > MIC for various dosing regimens.

Equation 1


(1) $$\%f\text{T}>\text{MIC}=[\text{T}-\text{Ln}\frac{\mathrm{R0}/\mathrm{CL}}{\mathrm{R0}/\mathrm{CL}-\mathrm{MIC}}\times \mathrm{Vd}/\mathrm{CL}$$

$$\;+\text{Ln}\frac{\mathrm{R0}/\mathrm{CL}-\mathrm{R0}/\mathrm{CL}\times e^{\left(-\mathrm{CL}/\mathrm{Vd}\times T\right)}}{\text{MIC}}\times \mathrm{Vd}/\mathrm{CL}]\times \frac{100}{\text{DI}}$$

The given equation includes several parameters as follows: The free drug fraction (*f*), natural logarithm (Ln), infusion rate (R0 = *f* × dose / T), renal clearance (CL, L/h), volume of distribution (Vd), minimum inhibitory concentration (MIC, μg/mL), intravenous infusion time (T, h), and dosing interval (DI, h). Clinical breakpoints for pathogen susceptibility are defined by the Clinical and Laboratory Standards Institute (CLSI) standards for meropenem (23). For *Enterobacterales*, MIC ≤ 1 μg/mL is considered susceptible, MIC = 2 μg/mL is considered intermediate, and MIC ≥ 4 μg/mL is considered resistant. For *Pseudomonas aeruginosa* and *Acinetobacter* spp., MIC ≤ 2 μg/mL is considered susceptible, MIC = 4 μg/mL is considered intermediate, and MIC ≥ 8 μg/mL is considered resistant (23).

### Population pharmacokinetic model and MIC distribution in critically ill patients

Monte Carlo simulations were performed using a population pharmacokinetic (PPK) model published by Gijsen et al. (24). Antimicrobial susceptibility testing of Gram-negative bacteria was derived from a total of 6,520 pathogens detected in critically ill patients in a tertiary care hospital (25). The pathogen-specific MIC distributions, which were used to calculate the cumulative fraction of response (CFR), are shown in Table 1.

**TABLE 1:** Pathogen-specific MIC distributions in critically ill patients (25).

	≤ 0.125	0.25	0.5	1	2	4	8	> 8
Overall (%)	82.7	4.7	3.5	2.1	1.3	1.3	1.0	3.3
*Acinetobacter baumannii*	8.4	33.7	21.7	7.2	3.6	1.2	0	24.1
*Pseudomonas aeruginosa*	12.8	20.6	20.5	13.2	6.2	7.5	5.9	13.3
*Klebsiella pneumoniae*	93.2	0.9	0.3	0.6	1.3	0.8	0.4	2.6

Antimicrob Agents Chemother, 2022. 66(2):e0183121. doi: 10.1128/AAC.01831-21. Originally published by and used with permission from American Association for Microbiolog.

### Optimized dosing regimens for critically ill patients with ARC treated with meropenem

Dosage regimens for meropenem were simulated in critically ill patients with CrCL of 140, 160, 180 and 200 mL/min. Suggested regimens included standard therapy, prolonged and continuous infusion. The CFR values were calculated as the weighted summation of the PTA values of each MIC category for a specific dosing regimen and renal clearance. Treatments with a CFR greater than 90% were considered as potential recommendations.

### Statistical analysis

Continuous variables are presented as mean (standard deviation) or median (quartiles), while categorical variables are presented as absolute numbers or relative frequencies. Statistical analyses were conducted using SPSS 22.0 software. A *p*-value of less than 0.05 was considered statistically significant.

## Results

### PTA of meropenem in critically ill patients with ARC

**FIGURE 1:**
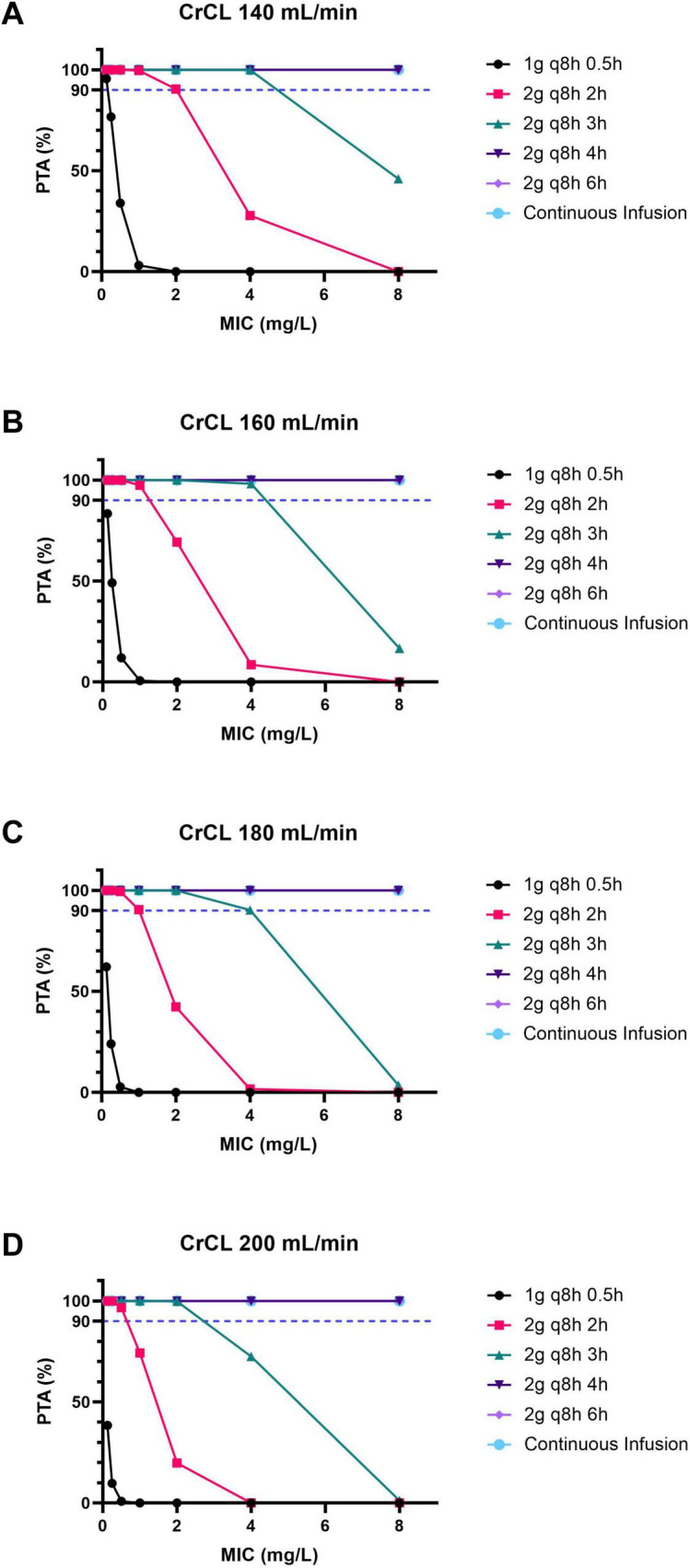
The PTA-MIC curves for patients with ARC under different meropenem dosing regimens. **(A)** For patients with a CrCL of 140 mL/min. **(B)** For patients with a CrCL of 160 mL/min. **(C)** For patients with a CrCL of 180 mL/min. **(D)** For patients with a CrCL of 200 mL/min. PTA, probability of target attainment; MIC, minimal inhibitory concentration; ARC, augmented renal clearance; CrCL, creatinine clearance.

Based on the present Monte Carlo simulations, for patients with a CrCL of 140 mL/min, the PTA-MIC curves of the meropenem regimens are shown in Figure 1A. For a target of 100% *f*T > MIC, for pathogens with MIC ≤ 2 mg/L, a PTA of 90% could be achieved with regimen b–f; for pathogens with 2 < MIC ≤ 4 mg/L, a PTA of at least 90% could be achieved with regimen c–f; for pathogens with 4 < MIC ≤ 8 mg/L, a PTA of at least 90% could be achieved with regimen d–f.

Likewise, for patients with a CrCL of 160 mL/min, the PTA-MIC curves of the meropenem regimens are shown in Figure 1B. For a target of 100% *f*T > MIC, for pathogens with MIC ≤ 1 mg/L, a PTA of 90% could be achieved with regimen b–f; for pathogens with 1 < MIC ≤ 4 mg/L, a PTA of at least 90% could be achieved with regimen c–f; for pathogens with 4 < MIC ≤ 8 mg/L, a PTA of at least 90% could be achieved with regimen d–f.

Furthermore, for patients with a CrCL of 180 mL/min, the PTA-MIC curves of the meropenem regimens are shown in Figure 1C. For a target of 100% *f*T > MIC, for pathogens with MIC ≤ 1 mg/L, a PTA of at least 90% could be achieved with regimen b–f; for pathogens with 1 < MIC ≤ 4 mg/L, a PTA of at least 90% could be achieved with regimen c–f; for pathogens with 4 < MIC ≤ 8 mg/L, a PTA of at least 90% could be achieved with regimen d–f.

Additionally, for patients with a CrCL of 200 mL/min, the PTA-MIC curves of the meropenem regimens are shown in Figure 1D. For a target of 100% fT > MIC, for pathogens with MIC ≤ 2 mg/L, a PTA of 90% could be achieved with regimen c–f; for pathogens with 2 < MIC ≤ 8 mg/L, a PTA of 90% could be achieved with regimen d–f.

### CFR of meropenem in critically ill patients with ARC

It is evident that, in order to achieve a target of 100% *f*T > MIC, a CFR of at least 90% can be attained in critically ill patients with ARC (140 ≤ CrCL ≤ 200 mL/min) when treated with regimens b, c, d, e, f. However, it is unfortunate that all CFR values in cases treated with meropenem 1 g q8 h 0.5 h are less than 90%, as illustrated in Table 2. Furthermore, for infections caused by *Acinetobacter baumannii* and *Pseudomonas aeruginosa*, the CFR target could not be achieved in patients with ARC. Furthermore, for infections caused by *Klebsiella pneumoniae*, the CFR target could be achieved in critically ill patients with ARC with regimens b, c, d, e, f.

**TABLE 2:** The CFR values for different renal functions and dosing regimens achieving a target of 100% *f*T > MIC.

CrCL (mL/min)	140	160	180	200
**Overall**				
1 g q8 h 0.5 h	83.91	71.71	52.60	32.25
2 g q8 h 2 h	94.53	93.95	93.35	92.56
2 g q8 h 3 h	96.06	95.74	95.51	95.25
2 g q8 h 4 h	96.60			
2 g q8 h 6 h	96.60			
Continuous infusion	96.60			
**Acinetobacter baumannii**				
1 g q8 h 0.5 h	41.47	26.19	13.92	6.69
2 g q8 h 2 h	74.56	73.38	71.71	69.09
2 g q8 h 3 h	75.80	75.78	75.68	75.46
2 g q8 h 4 h	75.80			
2 g q8 h 6 h	75.80			
Continuous infusion	75.80			
**Pseudomonas aeruginosa**				
1 g q8 h 0.5 h	35.41	23.33	13.47	7.10
2 g q8 h 2 h	74.74	71.66	68.45	64.22
2 g q8 h 3 h	83.50	81.64	80.28	78.79
2 g q8 h 4 h	86.70			
2 g q8 h 6 h	86.70			
Continuous infusion	86.70			
**Klebsiella pneumoniae**				
1 g q8 h 0.5 h	89.90	78.21	58.12	35.89
2 g q8 h 2 h	96.40	95.95	95.50	95.06
2 g q8 h 3 h	97.28	97.15	97.04	96.88
2 g q8 h 4 h	97.50			
2 g q8 h 6 h	97.50			
Continuous infusion	97.50			

### Dosing regimens and recommendation to real-world settings

The Monte Carlo simulation results indicate that meropenem alone is inadequate for achieving PK/PD targets in patients with ARC (140 ≤ CrCL ≤ 200 mL/min) for infections caused by *Acinetobacter baumannii* and *Pseudomonas aeruginosa*. It is recommended that treatment be switched to other susceptible drugs or combination therapy.

For infections caused by *Klebsiella pneumoniae*, the recommendations were delineated according to the MIC category in accordance with real-world settings (see Table 3). For resistant *Klebsiella pneumoniae* with an elevated MIC (4 < MIC ≤ 8), the administration of a prolonged infusion (i.e., 4 h, 6 h) or continuous infusion may be advantageous for patients with ARC.

**TABLE 3:** The recommended dosing regimens for meropenem in critically ill patients with ARC.

CrCL (mL/min)		140	160	180	200
*Acinetobacter baumannii*	The meropenem alone is insufficient to achieve PK/PD target. It is recommended to switch to other susceptible drug or combination therapy.				
*Pseudomonas aeruginosa*	The meropenem alone is insufficient to achieve PK/PD target. It is recommended to switch to other susceptible drug or combination therapy.				
*Klebsiella pneumoniae*	MIC ≤ 1	2 g q8 h 2 h	2 g q8 h 2 h	2 g q8 h 2 h	2 g q8 h 3 h
	1 < MIC ≤ 2	2 g q8 h 2 h	2 g q8 h 3 h	2 g q8 h 3 h	2 g q8 h 3 h
	2 < MIC ≤ 4	2 g q8 h 3 h	2 g q8 h 3 h	2 g q8 h 3 h	2 g q8 h 4 h
	4 < MIC ≤ 8	2 g q8 h 4 h / 6 h, continuous infusion			

## Discussion

In the present study, Monte Carlo simulations demonstrated that meropenem 2 g q8 h 2/3 h, administered to critically ill patients with ARC (CrCL 140–200 mL/min), achieved ≥ 90% PTA (100% fT > MIC) for lower MICs (≤ 2 mg/L). However, for higher MICs (4–8 mg/L), intensified regimens (2 g q8 h 4/6 h or continuous infusion) were required. The CFR analysis confirmed ≥ 90% target attainment for *Klebsiella pneumoniae* with regimens meropenem 2 g q8 h 2–6 h or continuous infusion, but not for *Acinetobacter baumannii* or *Pseudomonas aeruginosa*, regardless of regimen. For resistant *Klebsiella pneumoniae* (4 < MIC ≤ 8), prolonged (4–6 h) or continuous infusions are recommended. For *Acinetobacter baumannii* and *Pseudomonas aeruginosa*, alternative or combination therapies are advised due to insufficient PK/PD target attainment with meropenem monotherapy. These findings underscore the necessity for tailored dosing strategies in ARC patients, contingent on the pathogen’s MIC and renal function.

Sepsis has been defined as an acute, life-threatening condition caused by a dysregulated immune system response to infection (26), affecting millions of individuals annually and resulting in 1/6 ∼1/3 of those afflicted dying as a direct consequence (27). For adults exhibiting signs of septic shock, it is recommended that antimicrobials be administered promptly, ideally within one hour of recognition (28, 29). GNB represent the primary pathogens, accounting for at least 40% of pathogens associated with bloodstream infections (30). Given the alarming global spread of antimicrobial resistance represents a significant threat (5), the lack of appropriate antibiotics for severe infections becomes a crucial issue (31).

Meropenem, a carbapenem antibiotic, has a broad spectrum of antibacterial activity and is widely used in antimicrobial therapy for a variety of bacterial infections, particularly those caused by GNB (32). Meropenem exhibits a time-dependent bactericidal effect, whereby the efficacy of this antimicrobial against pathogens is determined by measuring the percentage of time that the unbounded drug concentration exceeds the MIC between doses. This indicator is also known as % *f*T > MIC (33). For critically ill patients, it has been established that the PK/PD targets for meropenem should be increased to 100% *f*T > MIC in order to achieve a higher survival rate and to minimize resistance development (14, 34). Therefore, in the present study, 100% *f*T > MIC were chosen as PK/PD targets for PTA assessment during Monte Carlo simulation. Nevertheless, Meropenem is a hydrophilic compound that is primarily excreted by the kidneys, which are highly susceptible to alterations in renal function (19). Likewise, a number of pathophysiological alterations can influence the pharmacokinetics of meropenem in critically ill patients, potentially increasing the probability of subtherapeutic levels and affecting the efficacy of therapeutic interventions (34).

Currently, ARC is defined as a urinary creatinine clearance of at least 130 mL/min/1.73 m^2^. The mechanism of ARC may be attributable to altered physiological processes in critically ill patients, leading to hyperdynamic and hypermetabolic states that increase cardiac output and renal blood flow. This, in turn, results in enhanced drug clearance through the kidneys (19). Furthermore, there is evidence that ARC development is associated with inflammatory stress response, fluid resuscitation, and the use of vasoactive drugs in critically ill patients (35). It has been demonstrated by research that neutropenia accompanied by fever is also a contributing factor to ARC (19). It has been observed that subtherapeutic levels of renally cleared drugs are present in patients who are undergoing ARC, for instance, the β-lactam antibiotics, aminoglycoside and vancomycin (35, 36). In such instances, a lack of sufficient therapeutic antibiotic concentration for patients with ARC has been linked to an increased incidence of treatment failure and the selection of more resistant pathogens (16, 37). Carlier et al. reported that the average % *f*T > MIC for piperacillin/tazobactam or meropenem was 61% for patients with ARC and 94% for non-ARC patients (20). Liebchen et al. reported that the patient with ARC exhibited inadequate serum trough levels despite meropenem infusion at the maximum approved dose (2 g every 8 h) (38). Therefore, therapeutic drug monitoring (TDM)-guided antibiotic dosing will hopefully maximize antibiotic exposure and reduce bacterial resistance (39), improving clinical outcomes of patients with ARC (38, 40, 41).

Standard dosing of antibiotics in intensive-care-unit (ICU) patients runs the risk of low serum concentrations due to altered physiological conditions such as ARC and increased volume of distribution (3). Low serum-concentrations in combination with multiresistant bacteria at a higher MIC lead to subtherapeutic antibiotic exposure, with the consequence of treatment failure and the selection of more resistant pathogens. As such, standard dosing would be an inadequate strategy in this setting (4). There are limited treatment options for MDR *A. baumannii* infections and inappropriate initial therapy is associated with increased mortality. Novel antibiotics and combination therapy of existing drugs are deemed necessary in this context (5).

Furthermore, for adults with severe infections, optimizing dosing strategies should be conducted in accordance with PK/PD principles and the specific pharmacological properties of the drug in question (29). A number of studies have been conducted to investigate the PPK of meropenem in critically ill patients (24, 42, 43). In the present study, we employed the PPK model proposed by Gijsen et al. and conducted Monte Carlo simulations to determine the optimal dosage of meropenem in critically ill patients with ARC (24). The PTA and CFR calculations have established the regimen recommendation.

In order to increase the percentage of *f*T > MIC, the efficacy and safety of prolonged and continuous infusion of meropenem and other β-lactams in critically ill patients, regardless of ARC, have been assessed (44). In a study of neutropenic children with ARC treated by meropenem or piperacillin, continuous infusion was found to reduce the inadequate antimicrobial exposure rate (8% vs. 85%) in comparison with intermittent infusion (45). The BLING-III randomized controlled trial demonstrated that the continuous infusion of meropenem is clinically superior to intermittent infusion in critically ill patients with sepsis (46). Abdul-Aziz et al. (47) have reported that prolonged infusion of β-lactam antibiotics yields reduced risks of 90-day and ICU mortality with increased clinical cures compared to intermittent infusions in patients with GNB infections. Dosing simulations suggest that using continuous infusion regimens may enhance bacterial killing (48). Furthermore, continuous infusion for critical orthotopic liver transplant recipients has been shown to minimize the risk of 30-day resistance (49). A comparative analysis of adverse event incidences, including neurotoxicity, cytopenias, and diarrhea, revealed no significant disparities between prolonged and intermittent infusion regimens (50). The total daily antibiotic dose for the continuous therapy was equivalent to those recommended for intermittent therapy (51). Conversely, a randomized clinical trial revealed that continuous administration of meropenem did not significantly decrease the all-cause mortality and emergence of pandrug-resistant or extensively drug-resistant bacteria at day 28 (52). The findings indicate a persistent uncertainty surrounding the efficacy of prolonged infusions of β-lactam antibiotics in enhancing clinical outcomes in critically ill adults with sepsis (53). The need for further research in this area is underscored by the necessity for large-scale prospective studies that can provide more definitive answers.

Not only does ARC, but also hypoalbuminemia, have the capacity to significantly alter the pharmacokinetics of antibiotics in critically ill patients (54, 55). An increase in the free fraction of drug resulting from hypoalbuminemia will lead to an increase in the Vd and a consequent increase in the rate of renal drug elimination (37). Hypoalbuminemia exerts a significant influence on highly albumin-bound (>90%) and predominantly renal eliminated antibiotics, such as ceftriaxone and ertapenem (56). The lower unbound fraction of vancomycin, along with lower binding antimicrobials, has been observed to be induced by ARC and hypoalbuminemia (57, 58). Consequently, alterations in septic shock, encompassing fluid overload, augmented cardiac output, ARC, and hypoalbuminemia, may result in subtherapeutic concentrations of antimicrobials, thereby influencing treatment efficacy and patient outcomes (59).

In light of that the predominant manifestation of bacterial infections occurs in extra-vascular tissues, the therapeutic effect of antibiotic treatment is contingent on the concentration of free antibiotic in target tissues (60, 61). For instance, respiratory tissue penetration of meropenem was reported to be 40% in the lung (62), and 37.5% of target site concentrations were below the EUCAST clinical breakpoint (63). This relatively low concentration in lung tissue may explain why achieving 50% *f*T > MIC does not necessarily improve clinical outcomes (64). Conversely, the plasma azithromycin concentrations were only approximately 10% and 1% those of bronchial fluid and lung tissue, respectively (65). Therefore, the free antibiotic concentrations in the tissues are responsible for the antibacterial activity and are more suitable for the determination of the clinical efficacy than the plasma concentration (66).

However, it should be noted that this study is subject to certain limitations. Firstly, the clinical trial is lacking, which means that there is no evidence to assess the efficacy of the regimen’s recommendation. Secondly, only one PPK model has been used in this study, which may not be the most appropriate model for Monte Carlo stimulation in critically ill patients with ARC. Therefore, further Monte Carlo stimulations and clinical studies are warranted.

In conclusion, Monte Carlo simulation analysis revealed that in critically ill patients with ARC (CrCL 140–200 mL/min), meropenem 2 g q8 h (2–3 h infusion) achieved optimal PTA for pathogens with MIC ≤ 2 mg/L. However, for more resistant organisms (MIC 4–8 mg/L), extended infusions (4–6 h) or continuous administration were necessary to maintain therapeutic efficacy. While these extended regimens proved effective against *Klebsiella pneumoniae*, they failed to achieve adequate coverage for *Acinetobacter baumannii* or *Pseudomonas aeruginosa* infections, highlighting the need for alternative antimicrobial agents or combination therapy approaches in such cases. These findings emphasize the importance of implementing individualized dosing strategies in ARC patients, taking into account meropenem’s unique PK/PD characteristics, including its time-dependent bactericidal activity and predominant renal elimination, to effectively manage resistant Gram-negative infections while optimizing clinical outcomes.

## Data Availability

The original contributions presented in this study are included in this article/supplementary material, further inquiries can be directed to the corresponding authors.
